# Outcomes for patients with head and neck squamous cell carcinoma presenting with N3 nodal disease

**DOI:** 10.1186/s41199-017-0027-z

**Published:** 2017-11-14

**Authors:** Matthew E. Witek, Aaron M. Wieland, Shuai Chen, Tabassum A. Kennedy, Craig R. Hullett, Evan Liang, Gregory K. Hartig, Randy J. Kimple, Paul M. Harari

**Affiliations:** 1 Department of Human Oncology, University of Wisconsin, 600 Highland Avenue, K4/B100-0600, Madison, WI, Madison, WI 53792 USA; 2 Department of Surgery, Division of Otolaryngology and Head and Neck Surgery, University of Wisconsin, Madison, WI USA; 3 Department of Biostatistics and Medical Informatics, University of Wisconsin, Madison, WI USA; 4 Department of Radiology, University of Wisconsin, Madison, WI USA; 5 University of Wisconsin School of Medicine and Public Health University of Wisconsin, Madison, WI USA

**Keywords:** Head and neck cancer, N3 nodal staging, Surgery, Radiation, Chemotherapy

## Abstract

**Background:**

The present study evaluated clinical outcomes for patients with head and neck squamous cell carcinoma presenting with N3 nodal disease.

**Methods:**

A retrospective analysis of N3 head and neck squamous cell carcinoma patients was performed. Pearson chi-square and Wilcoxon signed-rank tests were used to analyze patient demographics, disease characteristics, and treatment variables. Survival was evaluated using Kaplan-Meier curves with the log-rank test. Univariate analysis using Cox proportional hazards models was used to define factors associated with overall survival. Patient and tumor characteristics associated with treatment assignments were analyzed by univariate multinomial logistic regression.

**Results:**

We identified 36 patients with radiographically-defined N3 disease. For the entire cohort, median follow-up was 23.6 (range 2.8–135.0) months, and overall survival was 60% at 2 years and 30% at 5 years. Overall survival was similar between patients receiving primary surgery, radiotherapy, or chemoradiotherapy (*p* = 0.10). Primary, regional, and distant control at 5 years was 71%, 66%, and 53%, respectively. There was a trend towards improved regional control with primary surgery (*p* = 0.07). Planned neck dissection following primary chemoradiotherapy did not improve regional control (*p* = 0.55). Patients with p16-positive tumors exhibited improved overall (*p* = 0.05) and metastatic recurrence-free survival (*p* < 0.05). There were no factors predictive of treatment assignment nor factors associated with overall survival, local and regional control, or distant metastases free-survival on univariate analysis.

**Conclusions:**

Patients with N3 head and neck squamous cell carcinoma exhibit 5-year overall survival rates of approximately 30% regardless of treatment modality. Planned neck dissection does not improve regional control in patients undergoing definitive chemoradiotherapy. p16-positive patients represent a favorable cohort. Distant failure comprises the major failure pattern and should be the focus of future studies in improving the outcome of this patient cohort.

## Background

There is controversy regarding the optimum treatment of patients with head and neck squamous cell carcinoma and N3 nodal disease (N3 HNSCC). The variability in treatment approaches reflects the paucity of level one data to guide decision making as limited numbers of patients with N3 disease are included in randomized controlled trials meant to establish standards of care [1–6]. The rarity of inclusion of patients with N3 disease is notable in a recently completed randomized controlled trial in which only 11% of patients enrolled had N3 disease despite this being a stated goal of study accrual [3]. Clinical management of N3 HNSCC patients is typically influenced by case series that may reflect institutional bias, patient selection, and disease characteristics. Despite these confounders, the majority of data suggests that favorable rates of locoregional control can be achieved although there remains a high rate of distant metastatic disease approaching 40% [7–11].

The current study evaluates the long-term outcomes of patients with N3 HNSCC treated with either primary surgery, radiotherapy, or chemoradiotherapy approaches, compares treatment-specific toxicities, and evaluates the clinical impact of p16 status on N3 oropharyngeal squamous cell primary tumors.

## Methods

### Study population

Data collection for this retrospective analysis was approved by the University of Wisconsin-Madison Institutional Review Board. Patients included in the head and neck data base gave approval and written consent for usage of their information for research purposes. We identified 74 patients with N3 HNSCC treated at the University of Wisconsin-Madison from 1991 to 2015. Thirteen patients were excluded for having non-squamous cell histology, 3 for having distant metastatic disease at presentation, 3 for undergoing palliative intent treatment, 2 for having N2b disease, and 17 for not having cross-sectional imaging available for review. The final cohort consisted of 36 patients with either oral cavity, oropharyngeal, hypopharyngeal, laryngeal, or unknown primary squamous cell carcinoma with N3 disease (> 6 cm) as determined by radiographic assessment. Clinical records were reviewed in order to obtain patient characteristics, TNM classification, primary tumor site, radiographic nodal characteristics, p16 status (used as a surrogate for high-risk human papillomavirus (HPV) infection), primary and adjuvant treatment modalities, time to local, regional, and distant failures, and death.

### Statistical analysis

Standard descriptive statistics were used to analyze the distribution of covariates throughout the patient cohort. All of the baseline patient demographics and characteristics were analyzed using Pearson chi-square tests, and the Wilcoxon signed-rank test was used to test the continuous variables age, radiation dose, fraction size, and nodal volume. Survival was evaluated using Kaplan-Meier curves. The log-rank test was used to compare overall survival among the different treatment groups. Univariate analysis using Cox proportional hazards models was utilized to determine factors associated with overall survival. Univariate multinomial logistic regression was applied to patient and tumor characteristics to examine factors associated with treatment assignments. Statistical analyses were performed using *SAS 9.4 (SAS Institute Inc., Cary, NC).* All *p*-values were two-sided, and a *p* ≤ 0.05 was considered statistically significant for all analyses.

### Radiographic analysis

The computed tomographic images of each patient were retrospectively analyzed by a board certified neuroradiologist (TK) specializing in head and neck imaging. The largest abnormal nodal mass was identified in each patient and was measured in craniocaudal, anterior-posterior and transverse planes. Nodal volume was estimated by the ellipsoid calculation with which volume is approximated as half the product of the maximum dimensions in each axis (volume ≈ ½ *x X y X z*).

### Therapy

All patients included in this analysis had a performance status that permitted curative therapy. Treatment recommendations were made through group consensus at a Head and Neck tumor board attended by Head and Neck Surgery, Radiation Oncology, Medical Oncology, Radiology, and Pathology. Patients treated prior to 2001 received definitive radiotherapy (RT) given the standard of care of the time. Following 2001 patients typically received concurrent chemoradiotherapy (CRT) given randomized data supporting its superiority to RT alone [12]. RT was delivered by either Tomotherapy-based intensity modulated radiotherapy (IMRT) or 3D conformal technique using lateral photon fields supplemented with a matched low neck anterior-posterior photon field and nodal boosting with posterior neck electron fields. When given concurrently with chemotherapy, approximately 67% of patients received 70 Gy in 33 fractions of 2.12 Gy while the remaining 33% received 70 Gy in 35 fractions of 2 Gy. If chemotherapy was not utilized, radiotherapy was often given in BID (twice per day) fractions of 1.2–1.53 Gy to a total dose of 69.9–74.6 Gy. Varying low and intermediate risk dose and fractionation schemes approximating 54 Gy and 60 Gy, respectively, were used at the treating radiation oncologist’s discretion. Treatment volumes included gross disease and nodal volumes II, III, IV, and at the treating radiation oncologist’s discretion, lateral retropharyngeal lymph nodes, levels IB and V. The majority of patients received weekly concurrent cisplatin chemotherapy at 30 mg/m^2^. Cetuximab or cisplatin-docetaxel doublet were used occasionally. Radical, modified radical, or selective neck dissections were performed at the head and neck surgeon’s discretion.

## Results

### Patient, tumor, and treatment characteristics

We identified 36 patients with N3 HNSCC treated definitively from 1991 to 2015 at the University of Wisconsin-Madison that were followed for a median of 23.6 (range 2.8–135.0) months. Patient, tumor, and treatment characteristics are detailed in Table 1. Oropharyngeal tumors comprised 67% of the cohort representing the most common primary site. Of the 68% of oropharyngeal primaries that were stained for p16, 67% were positive. Unknown primary (11%), hypopharynx (11%), larynx (8%), and oral cavity (3%) were less common. Primary tumor stages were well represented. Median nodal volume was 42.7 (range 15.9–194.8) cm^3^ and was similar between all treatment cohorts (*p* = 0.88). Fifty-six percent of patients underwent primary CRT and 22% received either primary surgery or RT. Planned neck dissections were performed in 55% and 62% of patients receiving primary CRT or RT, respectively (*p* = 0.27).

**Table 1:** Baseline patient, disease, and treatment characteristics

	CRT (*n* = 20)	RT (*n* = 8)	Surgery (*n* = 8)	All (*n* = 36)	*p*-value
Age					0.51
Median	58.5	60.5	60	59	
(range)	(43–70)	(44–78)	(48–78)	(43–78)	
Sex					0.49
Male	18 (90.0%)	6 (75.0%)	6 (75.0%)	30 (83.3%)	
Race					0.86
Black	6 (30.0%)	3 (37.5%)	2 (25.0%)	11 (30.6%)	
White	14 (70.0%)	5 (62.5%)	6 (75.0%)	25 (69.4%)	
p16					0.64
Negative	2 (10.0%)	1 (12.5%)	2 (25.0%)	5 (13.9%)	
Positive	7 (35.0%)	1 (12.5%)	2 (25.0%)	10 (27.8%)	
Unknown	11 (55.0%)	6 (75.0%)	4 (50.0%)	21 (58.3%)	
Planned neck dissection of N3 neck					0.27
Yes	11 (55.0%)	5 (62.5%)	7 (87.5%)	23 (63.9%)	
Tumor Site					0.42
Hypopharynx	1 (5.0%)	2 (25.0%)	1 (12.5%)	4 (11.1%)	
Larynx	3 (15.0%)	0 (0.0%)	0 (0.0%)	3 (8.3%)	
Oral Cavity	0 (0.0%)	0 (0.0%)	1 (12.5%)	1 (2.8%)	
Oropharynx	14 (70.0%)	5 (62.5%)	5 (62.5%)	24 (66.7%)	
Unknown	2 (10.0%)	1 (12.5%)	1 (12.5%)	4 (11.1%)	
T-Stage					0.94
T0	2 (10.0%)	1 (12.5%)	1 (12.5%)	4 (11.1%)	
T1	3 (15.0%)	2 (25.0%)	3 (37.5%)	8 (22.2%)	
T2	5 (25.0%)	2 (25.0%)	1 (12.5%)	8 (22.2%)	
T3	6 (30.0%)	1 (12.5%)	0 (0.0%)	9 (25.0%)	
T4	4 (20.0%)	2 (25.0%)	1 (12.5%)	7 (19.4%)	
RT Type					< 0.01
IMRT	13 (65.0%)	0 (0.0%)	6 (75.0%)	19 (52.8%)	
Non-IMRT	7 (35.0%)	8 (100.0%)	2 (25.0%)	17 (47.2%)	
Fraction Size					< 0.01
Median	2.11	1.67	2.0	2.0	
(range)	(1.4–2.2)	(1.2–2.0)	(1.2–2.1)	(1.2–2.2)	
RT Total Dose					0.18
Median	7000 cGy	7010 cGy	6800 cGy	7000 cGy	
(range)	(6572–7320)	(6700–7460)	(6000–7440)	(6000–7460)	
Nodal Volume					0.88
Median	40.3	37.8	48.8	42.7	
(range)	(17.3–194.8)	(15.9–81.2)	(23.1–76.0)	(15.9–194.8)	

*Abbreviations*: *RT* radiotherapy, *CRT* chemoradiotherapy, *IMRT* intensity modulated Radiotherapy

### Treatment assignments

We evaluated patient and disease factors associated with receipt of treatment. Using surgery as the dependent variable, odds ratios were created and are shown in Table 2. In comparing RT with surgery and CRT with surgery, we were unable to identify any specific factor including age, T-stage, p16-status, and nodal volume that significantly predicted for treatment receipt.

**Table 2:** Factors associated with surgery of the primary tumor (Odds ratio > 1 indicates more likelihood of undergoing surgery)

	Odds Ratio (95% CI)	Odds Ratio (95% CI)
	Radiotherapy	Chemoradiotherapy
Age	1.01 (0.91–1.13)	0.95 (0.86–1.04)
T1	0.38 (0.02–6.35)	0.31 (0.02–4.02)
T2	1.00 (0.03–29.81)	1.25 (0.06–26.87)
T3	0.25 (0.01–7.45)	0.75 (0.05–11.31)
P16-negative	1.00 (0.03–29.81)	0.29 (0.02–3.52)
Nodal volume (cm^3^)	1.00 (0.96–1.03)	1.01 (0.98–1.03)

### Treatment outcomes

**Fig. 1:**
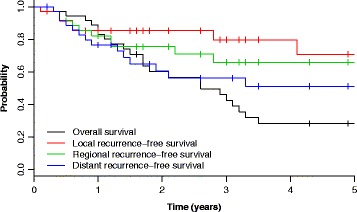
Clinical outcomes of patients with N3 NHSCC treated with either primary surgery (*n* = 8), radiotherapy (*n* = 8), or chemoradiotherapy (*n* = 20)

Clinical outcomes for the entire cohort are shown in Fig. 1. Overall survival at 2 and 5 years for the entire cohort was 60% and 30%, respectively. Local control was 86% and 71% and regional control was 77% and 66% at 2 and 5 years, respectively. Distant metastases disease free survival at 5 years was 53% (Fig. 1a-d).

**Fig. 2:**
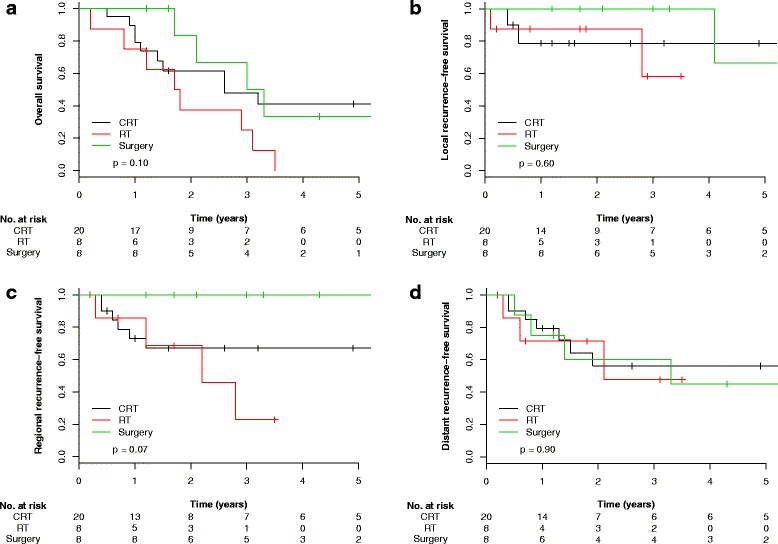
**a-d**. **a** Overall survival (*p* = 0.10) (**b**) local recurrence-free survival (*p* = 0.60) (c) regional recurrence-free survival (*p* = 0.07) and (**d**) metastasis recurrence-free survival (*p* = 0.90) of patients with N3 HNSCC treated with upfront surgery (*n* = 8), radiotherapy (*n* = 8), or chemoradiotherapy (*n* = 20)

**Fig. 3:**
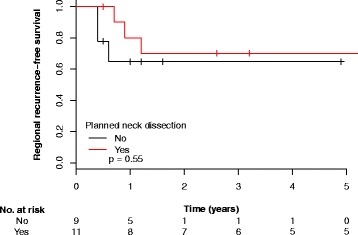
Regional recurrence of patients treated with primary chemoradiotherapy (*n* = 11) with or without (*n* = 9) planned neck dissection (*p* = 0.55)

**Fig. 4:**
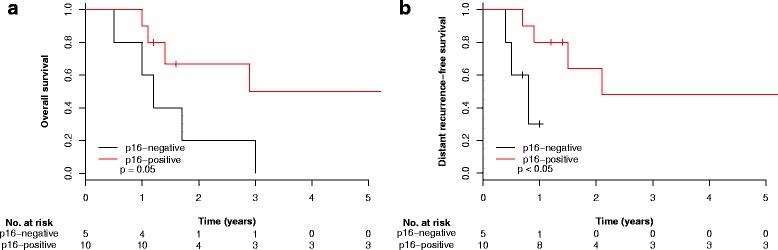
**a** and **b** Overall survival (**a**) (*p* = 0.05) and metastasis recurrence-free survival (*p* < 0.05) of patients with p16-negative (*n* = 5) and p16-positive oropharynx squamous cell carcinoma

Clinical outcomes were next evaluated in context of the primary treatment modality for either surgery, RT, or CRT. There were no statistically significant differences in overall survival, local-, regional-, and metastases-free survival (*p* = 0.10, *p* = 0.60, *p* = 0.07, *p* = 0.90, respectively) (Fig. 2a-d). Planned neck dissection did not impact regional recurrence-free survival following definitive CRT with approximately 70% being regionally controlled at 5 years (*p* = 0.55) (Fig. 3). Within the subset of patients with oropharyngeal primary tumors, p16 positivity conferred a survival advantage with 2-year overall survivals of 65% and 20% for p16-positive and p16-negative disease, respectively (*p* < 0.05) (Fig. 4a). Metastatic recurrence-free survival was also significantly different between patients with p16-positive and p16-negative oropharyngeal primaries (p < 0.05) (Fig. 4b).

The median time to the development of local, regional, and distant disease recurrences was 7.4 months (range 5.0–49.7), 9.7 months (range 3.1–40.0), and 13.8 (range 3.5–38.4) months, respectively. The majority of failures were distant metastases with lung and bone metastases being most common. Distant metastases were the most common cause of death (Table 3).

**Table 3:** Patient mortality

Cause of Death	Number (%)
Locoregional disease	6 (27)
Distant metastatic disease	9 (41)
Locoregional and distant metastatic disease	4 (18)
Intercurrent disease	2 (9)
Unknown	1 (5)

Salvage surgery was not performed for progressive nodal disease given unresectability in all cases. Salvage surgery for recurrent primary disease was performed on 2 patients. The remaining patients with locoregional or distant disease progression received palliative chemotherapy.

### Toxicity

Acute toxicities were similar between patients undergoing either primary surgery or radiotherapy except for grade 3 or higher mucositis, which was higher in patients treated with radiotherapy compared to surgery (81.3% versus 30.0%; *p* < 0.05). Sixty-eight percent of patients in the surgical and radiotherapy (68.4% v 68.0%; *p* = 0.98) groups required a feeding tube for a median of 6 months (range 2–42 months versus 3–33 months; *p* = 0.59). Neither treatment group had a patient with a permanent feeding tube requirement. Unplanned hospitalization within 6 months from diagnosis was similar between primary surgery and radiation groups (27.8% versus 36.0%; *p* = 0.57). There was no difference in weight loss between the groups with a median of approximately 12.5 kg measured from the beginning of the first treatment whether that being surgery, RT, or CRT to the end of treatment. Patients undergoing surgery as part of their care had various cranial nerves sacrificed with CN XI being most common occurring in 40% of patients.

## Discussion

Patients with N3 HNSCC disease comprise approximately 10% of subjects enrolled on randomized clinical trials. The applicability of the outcomes of these trials to patients with N3 HNSCC is therefore unclear. As such, the majority of data regarding management and outcomes of N3 HNSCC patients is derived from single institution studies. Along these lines, reported here is a retrospective analysis of 36 patients with N3 HNSCC treated at a single institution. The study demonstrates overall survival and regional control of 60% and 77% at 2 years and 30% and 66% at 5 years. Distant metastases were the predominant failure pattern occurring in 53% percent of patients at 5 years. These findings are congruous with those reported in other series.

Management of patients with N3 disease often reflects institutional patterns of care and disease characteristics. In that context, identifying treatment regimens that yield the highest therapeutic ratio of cure against morbidity is difficult. Despite these challenges, a recent study compared primary surgery to chemoradiotherapy and demonstrated improved outcomes in the surgical cohort with 5-year overall survival of 80% and 46% (*p* < 0.05) for surgery and radiotherapy, respectively [11]. In this study, we were unable to define a difference in any clinical outcomes when comparing patients undergoing primary surgery, RT, or CRT. The difference in conclusions may represent a lack of numerical power to detect a difference given the small sample size and/or patient selection differences. In the aforementioned study, 76% of patients in the primary surgery group were T0, T1, or T2 classified tumors while primary chemoradiotherapy was used for only 46% of similarly classified tumors. Further, all patients with nodal disease encasing the carotid or invading deep musculature underwent primary chemoradiotherapy. When this population of patients with more advanced regional disease was excluded from the primary radiotherapy group, the significant association between treatment modalities was lost (*p* = 0.07). In contrast, neither T-stage, p16-status, or nodal volume predicted for treatment assignment in the current study, which may have contributed to the non-significant result. Thus, larger studies with greater statistical power would be valuable, however in the absence of randomized controlled trials, the inherent selection bias that allocates patients to receive surgery vs non-surgical intervention as initial therapy will confound direct comparison of these treatment approaches on ultimate outcome.

Thirty to 80% of patients in N3 HNSCC series are comprised of oropharyngeal primaries [7–11, 13]. With data being collected from 1975 to 2010 there is likely considerable variability in the contribution of HPV-positive and HPV-negative tumors in these series. The prognostic implications of HPV status in patients with N3 disease has not been examined in N3 series. However, two recent studies evaluated the impact of tumor (T) and nodal (N) classification of HPV-positive oropharyngeal tumors. Interestingly, there was discordance between the studies regarding the impact of N3 disease. In the Princess Margaret study N3 status was considered a high-risk factor whereas in the MD Anderson analysis T4 but not N3 indicated high risk disease [14, 15]. Here, despite the small sample size, this study suggests that HPV likely confers a significant survival advantage for patients with oropharyngeal squamous cell carcinomas and N3 nodal disease as patients with p16-positive disease exhibited improved survival outcomes compared to those with p16-negative disease (*p* = 0.05). Interesting, local and regional control were similar between p16-postive and p16-negative patients whereas the incidence of distant metastases was significantly higher in the p16-negative cohort (*p* < 0.05). Given the small sample size it was not possible to evaluate the impact of treatment on p16-positive tumors.

Historically poor complete response rates to N3 nodes treated with primary RT lead to the practice of planned neck dissection. However, in the current era of CRT, clinical response of bulky adenopathy has improved [16, 17]. Analysis of Trans Tasman Radiation Oncology Group Study 98.02 demonstrated a zero incidence of isolated neck failures in patients that had a complete clinical and radiographic response [18]. More recently, Mehanna et al. demonstrated that post-CRT PET-CT-guided surveillance showed similar survival outcomes compared to planned neck dissection in those patients with N2 and N3 disease [19]. In our analysis, we found similar regional control rates in patients that underwent CRT alone with those that went on to receive planned neck dissection. Similar to the TROG data, we identified a single patient that had an isolated neck recurrence. Taken together, these data support observation in patients with complete clinical responses to CRT and suggest caution against planned neck dissections.

Chemotherapy given concurrently with radiotherapy improves outcomes in the primary and adjuvant setting [20]. Given the predominant distant metastatic pattern of failure in patients with N3 disease, a recent large randomized controlled trial evaluated the impact of induction chemotherapy followed by concurrent chemoradiotherapy in patients with advanced nodal disease. Despite the additional induction chemotherapy, there was no difference in distant failure-free or overall survival [3]. In our analysis, we did not identify a benefit in distant metastasis-free survival with the addition of chemotherapy. Given these data, newer approaches need to be explored to decrease or treat the development of metastatic disease.

This series contains several limitations inherent with retrospective analyses of an uncommon disease. First, the number of patients is small and treatment modalities varied considerably. Secondly, oropharyngeal cancers comprised a majority of the patients. Thus, it is not clear if the deduced outcomes for the overrepresented oropharyngeal tumors holds true for non-oropharyngeal cancers. Lastly, p16 data was available for a considerable portion, but not all, of our patient cohort. The majority were p16-positive, which could imply that the p16-negative cohort is not representative of the typical p16-negative patient.

## Conclusions

Several retrospective series have evaluated clinical outcomes for patient with N3 HNSCC disease. In each series, institutional practices have varied from primary radiotherapy with or without planned neck dissection to upfront surgical approaches. Despite such heterogeneous practice patterns and patient populations, reports have yielded consistent results supporting favorable locoregional control and unacceptable rates of distant failure. Although limited, existing studies suggest treatment recommendations and outcomes are heavily influence by disease presentation such as resectability [11] and patient characteristics [9]. As such, standards of care for such a rare disease will be difficult to establish given the difficulty in conducting randomized studies. Thus, larger database studies will help assess the most appropriate treatment modalities for N3 disease within particular primary tumor subsites and whether or not HPV positive patients with N3 nodes maintain a favorable prognosis. Currently, given long term survival of 30%, definitive treatment approaches should be pursed barred the presence of metastatic disease at diagnosis, while newer approaches at controlling systemic disease should be investigated.

## Abbreviations

BID: Twice per day
CN: Cranial nerve
CRT: Chemoradiotherapy
HNSCC: Head and neck squamous cell carcinoma
HPV: Human papillomavirus
IMRT: Intensity modulated radiotherapy
RT: Radiotherapy
